# Psychometric Testing of an Instrument Assessing Family Knowledge, Contemplation, Confidence and Readiness for Engaging in Early Mobilisation of Critically Ill Patients: A Multi‐Site Cross‐Sectional Design

**DOI:** 10.1111/jan.16415

**Published:** 2024-09-03

**Authors:** Sasithorn Mukpradab, Michael Ireland, Georgia Tobiano, Kristen Ranse, Fiona Coyer, Kellie Sosnowski, Daren K. Heyland, Andrea P. Marshall

**Affiliations:** ^1^ School of Nursing and Midwifery Griffith University Southport Queensland Australia; ^2^ Faculty of Nursing Prince of Songkla University Hat Yai Thailand; ^3^ Faculty of Sciences University of Southern Queensland Ipswich Queensland Australia; ^4^ Menzies Health Institute Queensland Griffith University Southport Queensland Australia; ^5^ Nursing and Midwifery Education and Research Unit Gold Coast Health Southport Queensland Australia; ^6^ Intensive Care Unit Princess Alexandra Hospital Woolloongabba Queensland Australia; ^7^ Intensive Care Unit Royal Brisbane and Women's Hospital Herston Queensland Australia; ^8^ The University of Queensland Saint Lucia Queensland Australia; ^9^ Intensive Care Unit Logan Hospital Meadowbrook Queensland Australia; ^10^ Queen's University Kingston Ontario Canada; ^11^ Kingston General Hospital Kingston Ontario Canada; ^12^ Intensive Care Unit Gold Coast University Hospital Southport Queensland Australia

**Keywords:** confirmatory factor analysis, critically ill patients, cross‐sectional study, early mobilisation, family engagement, psychometric testing

## Abstract

**Background:**

Early mobilisation is beneficial to support recovery among critically ill patients. The literature highlights the benefits of family engagement in early mobilisation, yet this practice remains underutilised. Effective implementation depends on understanding the key antecedents that influence family engagement in early mobilisation, specifically families' knowledge, contemplation, confidence and readiness. However, no measurement tools currently exist to assess these. Therefore, developing a psychometrically supported instrument is essential to understanding and enhancing families' factors influencing their engagement in early mobilisation.

**Aim:**

To develop and evaluate the psychometric properties of an instrument to assess families' knowledge, contemplation, confidence and readiness to engage in early mobilisation.

**Design:**

A multi‐site cross‐sectional survey design.

**Methods:**

Based on established psychological theory (Social Cognitive Theories and Behaviour Change Theories), an item pool was developed to assess families' knowledge, contemplation, confidence and readiness to participate in early mobilisation. To psychometrically evaluate the new tool, a multi‐site cross‐sectional survey was undertaken from May 2020 to June 2022 across five intensive care units in Australia. Data from 370 families of critically ill patients were used to evaluate the structural, convergent and discriminant validity as well as the reliability of the new instrument.

**Results:**

Confirmatory factor analysis indicated good model fit, supporting the proposed structure. All items displayed high standardised factor loadings except one, which improved upon freeing an error covariance. Positive inter‐factor correlations were moderate to strong and were substantially lower than the square root of the average variance extracted, supporting both convergent and discriminant validity, respectively. Additionally, all subscales demonstrated well to excellent reliability.

**Conclusion:**

The findings provide preliminary support for the multiple types of validity evidence and the reliability of the instrument. This new instrument is suitable for use in clinical and research applications to assess families' knowledge, contemplation, confidence and readiness for their engagement in early mobilisation.

**Impact:**

- Family engagement in early mobilisation activities may have multiple benefits but it is not commonly implemented in the ICU.
- Factors influencing family engagement in early mobilisation are poorly understood. Influential psychological theories highlight the likely importance of knowledge, contemplation, confidence and readiness. A readily available instrument designed to assess these constructs among family members is needed to deepen research understanding and guide clinical practice.
- The proposed instrument is designed to measure factors influencing family engagement in early mobilisation, which may support healthcare professionals and health services to identify and tailor strategies to support family engagement in early mobilisation.

**Reporting Method:**

Recommendations for reporting the results of studies of instrument and scale development and testing was followed to report this study.

**Patient or Public Contribution:**

Family members of adult critically ill patients participated in this study, and they provided the data through the survey.

**Summary**

**What Does This Paper Contribute to the Wider Global Clinical Community?**

- Preliminary psychometric support for the instrument supports its use in evaluating the role played by family members' knowledge, contemplation, confidence and readiness regarding family engagement in early mobilisation for adult critically ill patients.
- This instrument could be used in further research to understand family member knowledge, contemplation, confidence and readiness for their engagement in early mobilisation, and these data could be used to improve the practice of this approach across ICU settings.

## 1 Introduction

Critically ill patients often have acute organ failure and require admission to the intensive care unit (ICU) for advanced life‐sustaining treatment (Maslove et al. 2022). During critical illness, patients can experience prolonged periods of bed rest that can lead to the development of complications including ICU acquired weakness (Vanhorebeek, Latronico, and Van den Berghe 2020). Although promoting early mobilisation throughout ICU stay is a well‐established strategy for improving physical function, barriers to early mobilisation may limit how frequently this occurs (Anekwe et al. 2020). Several strategies have been recommended to improve the practice of early mobilisation, such as using guidelines/protocols, providing staff education and training, identifying clinical champions, bolstering physiotherapist staffing and providing more mobility equipment (Anekwe et al. 2020). However, early mobilisation may still not be sustainably enacted despite these strategies. A collaborative approach, actively partnering with family members as advocates and participants, may enhance previously suggested strategies (Anekwe et al. 2020) and complement existing team‐based approaches (Haines 2018; Mukpradab, Mitchell, and Marshall 2022; van Delft et al. 2021).

### 1.1 Background

Engaging family members in care during hospitalisation is an evidence‐based practice with proven benefits for patient recovery and healthcare quality (Park et al. 2018; Wiles et al. 2022). In the context of ICU, family members are increasingly considered as partners in decision‐making and care activities because they know the patient the best (Burns et al. 2018; Marshall et al. 2023). When family members engaged in patient care, there can be positive outcomes for patients, healthcare professionals and family members (Goldfarb et al. 2017; Marshall et al. 2017). A systematic review of the effect of family engagement interventions on outcomes found reduced ICU length of stay and increased patient‐and‐family satisfaction (Goldfarb et al. 2017). Family engagement interventions were also found to increase family members' comprehension of the patient's clinical situation and this reduced negative impacts on family members' mental health (Goldfarb et al. 2022). Consequently, engaging family members in early mobilisation in the ICU is a promising strategy to improve outcomes for patients.

A growing body of evidence demonstrates the positive influence of families partnering with healthcare professionals to promote early mobilisation in the ICU. Specifically, Rukstele and Gagnon (2013) reporting that the use of family engagement in early mobilisation increased the rate of early mobilisation practice from 66% to 94%. This engagement also reduced family members' stress, which was attributed to them being able to observe patient progress (van Delft et al. 2021). Feeling helpful and more connected with the healthcare team was also identified as a benefit (Felten‐Barentsz et al. 2021). Healthcare professionals also reported benefits and reported feeling more satisfied with the quality of care and the achievement of mobility and rehabilitation goals (van Delft et al. 2021).

Despite the emerging evidence demonstrating the benefits of family engagement in early mobilisation of adult critically ill patients, family members are not all the same and some may be more ready to engage than others (Ågård et al. 2019; Felten‐Barentsz et al. 2021; Haines 2018). In a longitudinal qualitative study, 10 family members of critically ill patients found that most family members lacked confidence in engaging in early mobilisation because they did not have knowledge regarding physical exercise activities (Felten‐Barentsz et al. 2021). Additionally, family members recognised that the patient's health‐changing conditions made them vulnerable, and they were also fearful of inadvertently harming the patient (Felten‐Barentsz et al. 2021). Nevertheless, research suggests that most family members want to actively assist their loved ones with regaining physical function if they feel confident in their understanding and ability to actively participate in early mobilisation (Felten‐Barentsz et al. 2021). Family members will also need personalised strategies to support their engagement in early mobilisation which requires evaluation of their readiness to engage in this activity. As yet, measures to assess family members' knowledge, contemplation, confidence, and readiness in engaging in early mobilisation are lacking. Such assessments are needed to provide healthcare professionals with a comprehensive understanding of family members' readiness to engage in early mobilisation activities, thereby facilitating the development of tailored strategies to enhance the successful implementation of family engagement in early mobilisation in the ICU (Felten‐Barentsz et al. 2021; Haines 2018; van Delft et al. 2021).

### 1.2 Instrument Development

Social Cognitive Theories (SCTs) and process models (Stages of Change Model and the Theory of Planned Behaviour) offer comprehensive, multi‐faceted frameworks as theoretical grounding for developing our instrument to understand family members' factors in supporting their engagement in early mobilisation in ICU settings (Bandura 1986; Prochaska and DiClemente 1983). For SCTs, this influential framework provides an account of factors influencing family members' willingness and ability to engage in early mobilisation. Its emphasis on the dynamic interplay of personal beliefs, environmental factors, and behavioural outcomes aligns directly with our developed instrument's focus on knowledge, contemplation, self‐efficacy and readiness.

Social Cognitive Theories also highlights how anticipated outcomes influence behaviour (outcome expectations). Knowledge is foundational—understanding the potential benefits of early mobilisation creates positive outcome expectations. For example, items such as *‘How well informed are you about the harms of inactivity and bed rest?’* help determine whether family members have the information necessary to envision positive results from their involvement. Furthermore, SCTs emphasises how individuals learn by observing others (observational learning). In the ICU, family members witness the actions and experiences of patients and other family members. Items assessing their understanding of *‘The physical function and activity level of your family member during the current hospitalisation?’* and their observations of *‘The various mobility and rehabilitation treatment options in general?’* shape their understanding of the care process. This observational learning impacts their expectations of potential outcomes and their sense of agency within this unique environment (Bandura 1986).

Regarding self‐efficacy which is a central component of SCTs, Bandura (1977) described that self‐efficacy is the belief in one's ability to perform specific tasks. By including items like *‘How confident are you that today you could participate in the mobility activity of your family member while in the hospital?’* and ‘*How confident are you that today you could seek out additional information on mobility and rehabilitation as it relates to the care of your family member?’*, the instrument measures this critical predictor of proactive engagement. Strong self‐efficacy allows family members to see themselves not just as passive recipients of care, but as active partners contributing positively to their loved one's recovery.

In terms of Process Models: Stages of Change and Theory of Planned Behaviour, focusing on knowledge, contemplation, self‐efficacy and readiness aligns with several influential behaviour change theories, notably the Stages of Change Model (Transtheoretical Model) (Prochaska and DiClemente 1983) and the Theory of Planned Behaviour (Ajzen 1991). Stages of Change Model describes a process of behaviour change where individuals progress through stages such as precontemplation, contemplation, preparation, action and maintenance. Our developed instrument assesses the contemplation stage, encouraging reflection on engagement, by including items like *‘How much have you thought about participating in the mobility and rehabilitation care of your family member while in the hospital?’* and *‘How much have you thought about the importance of mobility and rehabilitation in the hospital?’*. These questions gauge a family member's current level of consideration. Similarly, measuring readiness with questions like *‘How ready are you to participate in the mobility activity of your family member in while in the hospital?’* and *‘How ready are you to talk with a therapist and ask questions about the mobility and rehabilitation care of your family member?’* directly taps into the preparation stage, gauging their proximity to taking action (Prochaska and DiClemente 1983).

Moreover, the Theory of Planned Behaviour predicts behaviour through the complex interplay of attitude, subjective norms and perceived behavioural control. Items assessing knowledge (e.g., *‘How well informed are you about the mobility and rehabilitation care plan for your family member?’*) and contemplation (e.g., *‘How much have you thought about the importance of mobility and rehabilitation in the hospital?’*) influence a family member's overall attitude towards early mobilisation. Their sense of self‐efficacy (e.g., *‘How confident are you that today you could ask questions of the doctor regarding the mobility and rehabilitation care of your family member?’*) contributes to perceived behavioural control, a significant driver of intention to act (Ajzen 1991). Finally, while less explicit, our instrument also includes a couple of items that touch on social expectations or support, thereby recognising the influence of subjective norms on family behaviour. These items, like *‘How much have you thought about asking questions of the nurse about the mobility and rehabilitation care of your family member?’* and *‘How ready are you to talk with a therapist and ask questions about the mobility and rehabilitation care of your family member?’*, promote reflection on expectations and social support within their decision‐making process.

This theoretically grounded approach for developing our instrument offers several distinct advantages. By aligning the subscales of the instrument with key SCTs constructs, Stages of Change and the Theory of Planned Behaviour, it offers a comprehensive and rich understanding of the interconnected family members' knowledge, contemplation, confidence (self‐efficacy) and readiness which can influence their engagement in early mobilisation. This goes far beyond simply measuring attitudes or beliefs; it allows for the identification of specific points where interventions can have the most meaningful impact. Furthermore, drawing upon these robust theoretical frameworks provides a shared language for clinicians and researchers, facilitating effective communication and collaboration when implementing support strategies. Measuring these factors offers valuable insights into how family members internalise information and perceive their role, providing a basis for strategies that address specific misconceptions or uncertainties. Consequently, our developed instrument's theoretical foundation ensures that interventions are not only practical but also designed to target the core drivers of change, increasing the likelihood of sustained behavioural shifts and positive outcomes for both patients and their family members. This instrument not only identifies gaps in family members' knowledge, contemplation, confidence and readiness but also provides a roadmap for healthcare teams to bridge these gaps through targeted education and involvement strategies.

### 1.3 Aim

The aim of this study was to psychometrically evaluate a newly developed instrument developed to assess family members' knowledge, contemplation, confidence and readiness for family engagement in early mobilisation of critically ill patients.

## 2 Methods

### 2.1 Study Design

A multi‐site cross‐sectional survey design was used to evaluate evidence for the validity and reliability of the instrument. Recommendations for reporting the results of studies of instrument and scale development and testing was followed to report this study (Streiner and Kottner 2014).

### 2.2 Setting

This study was undertaken from May 2020 to June 2022 in the ICUs of five hospitals in southeast Queensland, Australia. Bed numbers ranged from five to 36. All ICUs provided high‐level intensive care treatment for adult critically ill patients. Annual admissions for each ICU ranged from 450 to 2300 patients. Each ICU was staffed by multidisciplinary teams of healthcare professionals responsible for early mobilisation of critically ill patients including physicians, nurses and physiotherapists. The nurse–patient ratio was one‐to‐one for patients receiving mechanical ventilation and one‐to‐two for stable non‐ventilated patients. The ICUs had open visiting with maximum two visitors at the bedside at any given time. In general, family members were not present for approximately 1 h during an established time for handover and the initial multidisciplinary round in daily morning. For some times during the data collection period, restricted family visiting was in place owing to the global pandemic of coronavirus disease 2019 (COVID‐19). Although healthcare professionals supported early mobilisation practice, family members were not commonly engaged in early mobilisation activities within each ICU.

### 2.3 Participants, Recruitment and Sample Size

This study considered the concept of family members to be broad and included both relatives and/or close friends who were involved in the ongoing care of, or provided support to the patient who was critically ill. Inclusion criteria stipulated respondents be family members who were (i) aged more than 18 years, (ii) expected to visit regularly (minimum three times a week) while the patient was in hospital and (iii) able to communicate in English both verbally and in writing. As our focus was on family engagement in an adult critical care context, we only recruited family members of critically ill patients who were over 18 years of age. We excluded any family members of patients who were not expected to stay in an ICU for at least 72 h or who were not expected to survive the ICU admission.

Research assistants from each of the participating ICUs identified potential participants by screening patients and family members according to above eligibility criteria. Family members were approached approximately 48 h before ICU discharge and provided a written and verbal description of the research study. The research assistant then returned to potential participants at an agreed time to answer any questions and determine their interest in voluntary participation.

Minimum sample size recommendations for psychometric testing vary in the literature (Pett, Lackey, and Sullivan 2003). Most guidelines endorse a participant to item ratio of 10:1 as acceptable for psychometric testing (Cabrera‐Nguyen 2010) and we aimed for this ratio to ensure robust analyses. The instrument had 22 items; however, we anticipated incomplete surveys as well as some with invalid responses. As recruitment was occurring amidst the COVID‐19 pandemic, we considered that incomplete and/or invalid surveys might be higher than usual so we intentionally overrecruited, aiming for 330 returned surveys.

### 2.4 Instrument

With an aim of balancing theoretical grounding with practical and clinical applicability, the instrument items were developed based on the team's expert knowledge of the literature, clinical experience and previous experience conducting research with family members in ICU. Building on the theoretical foundation outlined in the introduction, SCTs and process models of behaviour change (Stages of Change Model and Theory of Planned Behaviour) guided the selection of constructs and informed item wording (Ajzen 1991; Bandura 1977; Prochaska and DiClemente 1983). User feedback and content validity were then assessed, respectively, through consultation with consumers (including patients and family representatives with experience of ICU settings) and healthcare professionals with extensive experience in ICU and family engagement in care. Feedback was gathered on item clarity, ease of completion and the time to complete the survey. Minor refinements were made based on this feedback. The final instrument included 22 items assessing family members' (i) knowledge (six items), (ii) contemplation (six items), (iii) self‐efficacy (five items) and (iv) readiness (five items). While all items used a five‐point Likert scale, the specific semantic anchors were tailored to best fit the phrasing of items for each construct. The instrument is shown in File S1.

### 2.5 Data Collection

To facilitate timely completion, we encouraged family members to complete the instrument while visiting the ICU or while they were in the waiting room. We considered completing the instrument on a smart phone to be challenging and we were not permitted to share other electronic devices owing to the COVID‐19 pandemic. Consequently, we used a paper‐based survey to collect the data. This method has been reported to be more effective than electronic surveys (Beatty et al. 2020). In addition to completing the instrument, eligible family members who expressed interest in participating in the study were also asked to provide demographic data including age, gender, relationship to patient, whether the family member lived with the patient and level of education. Each family member spent approximately 10–15 min to complete the survey and the completed documents were returned to the research assistant from each ICU.

### 2.6 Data Analysis

Data were entered into Research Electronic Data Capture (REDCap) (Harris et al. 2009) and exported into IBM SPSS Statistics for Windows Version 20 (IBM Corp, Armonk, NY). Prior to analysis, data were cleaned by (i) cross‐checking the accuracy of entered data with a random 10% of the paper surveys, (ii) identifying the variable names and (iii) assessing the proportion of missing data. Although we allowed participants to indicate if they were unsure about any of the items, for the purpose of scoring the scales and data analysis (given *‘not sure’* is not part of the underlying theoretical continuum being measured), this response was recoded and treated as missing data. Data imputation methods were not used for missing data because the overall proportion of missing data was less than 10% (Bennett 2001).

Initial data analysis began with an assessment of data distribution, specifically skewness and kurtosis. Descriptive statistics were used to analyse demographic data. Normally distributed data, such as age, was analysed using mean and standard deviation. Categorical data, such as gender, relationship to patient, whether the family member lived with the patient, level of education and the response distribution to each item of the instrument were reported with frequency distributions and percentages (Polit‐O'Hara and Beck 2014).

Psychometric analysis was conducted using Mplus (version 8) (Muthen and Muthen 2017). As the subscales and items of the instrument were specified a priori, and therefore, its expected structure was known, confirmatory factor analysis (CFA) was the strictest method to test whether the items adequately fit the theoretical specification (Hair, Babin, and Anderson 2018). Our analyses assumed that if the instrument has adequate conceptual structure and robust organisation, the confirmatory model would yield good fit to the confirmatory model and exhibit the factor loadings (Hair, Babin, and Anderson 2018).

As ordered categorical data, Likert responses are not continuous, and therefore, the Weighted Least Square Mean and Variance (WLSMV) estimation method was used as it was specifically designed for this application. This WLSMV estimator makes no distributional assumptions about the observed variables (Li 2016). Multiple indicators of model fit were used with well‐established cut‐offs applied (Hair, Babin, and Anderson 2018). Specifically, normed Chi‐Square (*χ* ^2^/degree of freedom [df]) ≤ 3, Comparative Fit Index (CFI) and Tucker Lewis Index (TLI) ≥ 0.95, Root Mean Square Error of Approximation (RMSEA) ≤ 0.06 and Standardised Root Mean Square Residual (SRMR) ≤ 0.08 (Hair, Babin, and Anderson 2018). For completeness, the inferential Chi‐square (*χ* ^2^) test is reported with accompanying *p*‐value though it is not relied upon to determine model fit due to its known limitations in the context of larger samples and complex models (Hair, Babin, and Anderson 2018). The analysis also examined standardised factor loadings, Average Variance Extracted (AVE) and inter‐factor correlations. Given the theoretical links between the constructs being measured, significant positive correlations provide evidence of convergent validity. AVE values exceeding 0.50 indicate acceptable convergent validity, by showing that the latent construct is well‐represented by its observed indicators, capturing over 50% of their variance (Fornell and Larcker 1981). Finally, AVE values substantially higher than inter‐factor correlations also provide evidence of discriminant validity (Fornell and Larcker 1981).

Ordinal reliability coefficients, specifically Omega (*ω*), were calculated to assess internal consistency. Omega is specifically tailored for ordinal data, as it does not rely on assumptions of normality or equal intervals and is robust against outliers and skewed distributions. Conventional benchmarks for interpreting Omega coefficients include >0.70 for ‘adequate’, >0.80 for ‘good’ and >0.90 for ‘excellent’ internal consistency (Dunn, Baguley, and Brunsden 2014). Additionally, we calculated composite reliability (CR) estimates from CFA. CR, an internal consistency metric, accounts for measurement error by considering the standardised factor loadings of items. While CR may be inflated with low item numbers, it is widely regarded as a reliable measure of internal consistency, particularly when items have diverse factor loadings or differing relationships with the construct (Raykov 1997). Benchmarks for interpreting Omega coefficients include >0.60 for ‘adequate’, >0.70 for ‘good’, and >0.80 for ‘excellent’ internal consistency (Hair, Babin, and Anderson 2018).

### 2.7 Ethics Statement

The study was approved by the Human Research Ethics Committee at Griffith University (GU2020/047) and Gold Coast Health (HREC/2019/QGC/56845) which allowed for ethical approval across all sites under Australia's National Mutual Acceptance Scheme. Each site was also required to complete a site‐specific assessment.

## 3 Results

In total, 785 family members of adult critically ill patients were approached to participate in this study. Of these 373 returned the survey (response rate = 47.52%). Three surveys were excluded from the data analysis as they had missing data on all question items; therefore, 370 completed surveys (completion rate = 99.20%) were included in the data analysis. The total rate of missing data ranged from 2.68% to 9.12% inclusive of those questions to which participants responded, ‘not sure’ (range from 1.07% to 6.70%) and un‐answered items (range 1.34% to 3.49%).

### 3.1 Characteristics of Participants

The majority of participants were female (71.35%, *n* = 264) and had a mean age of 51.53 years (SD = 14.47 years) with the age range from 19 to 90 years. More than half of family members lived with the patients and 42.43% of participants were spouses or partners of patients. The majority of respondents who were living with patients were also those who were spouses or partners. There was variability in levels of education, and more than three‐quarters of family members reported their health to be good to excellent. The characteristics of family members of adult critically ill patients are shown in Table 1.

**TABLE 1:** Characteristics of family members (*N* = 370).

Variables	Descriptor	*n* (%)
Gender	Female	264 (71.35)
	Male	98 (26.49)
Relationship to patient	Spouse/Partner	157 (42.43)
	Parent/Parent‐in‐law	59 (15.95)
	Daughter/Son	96 (25.94)
	Sister/Brother	29 (7.84)
Living with the patient	Yes	202 (54.59)
	No	161 (43.51)
	More than weekly for patient contacts	55 (34.16)
	Weekly for patient contacts	42 (26.09)
	Monthly for patient contacts	33 (20.50)
	Yearly for patient contacts	17 (10.56)
	Less than once a year for patient contacts	5 (3.11)
Levels of education	Primary school or less	2 (0.54)
	Some high school	55 (14.86)
	High school graduate	98 (26.49)
	Some college or university	99 (26.77)
	University degree	72 (19.46)
	Post Graduate university degree	36 (9.73)
Health levels	Excellent to good	322 (87.03)
	Fair	26 (7.03)
	Poor	2 (0.54)

### 3.2 Confirmatory Factor Analysis

The four‐factor structures for the 22 item of the instrument demonstrated strong model fit across descriptive indices (*χ* ^2^/df = 2.48; CFI = 0.99; TLI = 0.99; RMSEA = 0.06 and SRMR = 0.05). Whereas the inferential *χ* ^2^ test was significant (*χ* ^2^ = 503.37, df = 203, *p* < 0.001). All but one standardised factor loading was high and statistically significant (*p* < 0.05), with only item one of the knowledge factor slightly below the below pre‐determined cut‐off. Factor loadings before the model modification were presented in File S2.

The CFA model, based on a priori theoretical assumptions, demonstrated satisfactory fit without modification, confirming the structural validity of the proposed factor structure. However, recognising that CFA models can benefit from further refinement, a supplementary exploratory analysis was conducted. The modification indices identified a high value for freeing the covariance between the error terms of items one and two of the knowledge factor. This finding aligns with a theoretical rationale rooted in the potential for method variance to influence these items. Specifically, both items assess the family member's physical function, one pre‐hospitalisation (item 1) and the other during hospitalisation (item 2). Theoretically, it is plausible that respondents may rely on similar cognitive processes or reference points when answering these questions, leading to shared variance beyond the underlying ‘knowledge’ factor. This shared variance could also be attributed to method variance, where the measurement method itself (i.e., the similar wording and focus of the items) introduces an additional source of correlation.

Allowing these error terms to correlate is a way to account for this method variance. In doing so, the model fit improved negligibly. The chi‐square difference test revealed a decrease in chi‐square value (Δ*χ* ^2^ = 9.8, Δdf = 1, *p* = 0.0017). While this decrease is significant at the conventional alpha level of 0.05, it does not reach significance at the more conservative level of 0.001 often used in CFA (Hu and Bentler 1999). The primary fit indices remained excellent both before and after modification (*χ* ^2^/df = 2.42; CFI = 0.99; TLI = 0.99; RMSEA = 0.06 and SRMR = 0.05) although the inferential *χ* ^2^ remained significant (*χ* ^2^ = 493.57, df = 204, *p* < 0.001). Of note, the standardised factor loading for item one increased from 0.49 to 0.54, while the loadings for all other items remained high and statistically significant (Table 2). The AVE for each set of subscale items also exceeded 0.50, both before and after the modification (Table 2).

**TABLE 2:** The standardised factor loading for each item and AVE for each factor (*N* = 370).

Items	Factor loading	AVE
**Knowledge**		
*How well informed are you about*…		
…The overall physical function of your family member prior to hospitalisation (i.e., walking ability, activity level, etc.)?	0.54	0.64
…The physical function and activity level of your family member during the current hospitalisation?	0.64	
…The harms of inactivity and bed rest?	0.82	
…The various mobility and rehabilitation treatment options in general? (Exercises, sitting out of bed, balance training, thinking activities, orientation, breathing exercises, standing and walking)?	0.89	
…The mobility and rehabilitation care plan for your family member?	0.91	
…The questions to ask about the mobility and rehabilitation care provided to your family member?	0.91	
**Contemplation**		
*How much have you thought about*…		
…The importance of mobility and rehabilitation in the hospital?	0.81	0.78
…Seeking additional information on mobility and rehabilitation as it relates to the care of your family member?	0.89	
…Asking questions of the therapists about the mobility and rehabilitation care of your family member?	0.96	
…Asking questions of the nurse about the mobility and rehabilitation care of your family member?	0.94	
…Asking questions of the doctor about the mobility and rehabilitation care of your family member?	0.90	
…Participating in the mobility and rehabilitation care of your family member while in the hospital?	0.78	
**Confidence (self‐efficacy)**		
*How confident are you that today you could*…		
…Seek out additional information on mobility and rehabilitation as it relates to the care of your family member?	0.93	0.83
…Ask questions of the therapist regarding the mobility and rehabilitation care of your family member?	0.95	
…Ask questions of the nurse regarding the mobility and rehabilitation care of your family member?	0.97	
…Ask questions of the doctor regarding the mobility and rehabilitation care of your family member?	0.93	
…Participate in the mobility activity of your family member while in the hospital?	0.76	
**Readiness**		
*How ready are you to*…		
…Seek out additional information on mobility and rehabilitation as it relates to the care of your family member?	0.88	0.71
…Talk with a therapist and ask questions about the mobility and rehabilitation care of your family member?	0.90	
…Talk with a nurse and ask questions about the mobility and rehabilitation care of your family member?	0.88	
…Talk with a doctor and ask questions about the mobility and rehabilitation care of your family member?	0.81	
…Participate in the mobility activity of your family member in while in the hospital?	0.74	

While this exploratory modification provides additional insights into potential sources of shared variance, it is important to emphasise that the original, unmodified model already demonstrated satisfactory fit and confirmed the hypothesised factor structure. The exploration of method variance serves to further refine our understanding of the data and inform future scale development.

### 3.3 Reliability: The Internal Consistency and Composite Reliability

Knowledge, contemplation, confidence and readiness factors had good‐to‐excellent internal consistency reliability at *ω* = 0.85, 0.92, 0.91 and 0.88, respectively. The CR for the subscales of knowledge, contemplation, confidence and readiness at 0.92, 0.97, 0.96 and 0.93, respectively.

### 3.4 Inter‐Factor Correlations: Converging and Discriminant Validity

The inter‐factor correlations were all statistically significant (*p* < 0.001), positive and moderate to strong (*r*s = 0.43–0.58; see Table 3). The direction and magnitude of the inter‐factor correlations suggest the factors are related but not so strongly as to be indistinguishable, thus providing support for the convergent validity of the instrument. Furthermore, the inter‐factor correlations fall below the range considered problematic for discriminant validity which is higher than 0.85 (Brown 2015), suggesting the factors are sufficiently distinct and supporting discriminant validity.

**TABLE 3:** Inter‐factor correlation matrix with square roots of AVE estimates in the diagonals.

Factor	Knowledge	Contemplation	Confidence	Readiness
Knowledge	0.80	0.43	0.58	0.51
Contemplation		0.88	0.40	0.50
Confidence			0.91	0.49
Readiness				0.84

Similarly, the square root of the AVE estimates (average square root of AVE = 0.86) is substantially higher for each factor than the bivariate correlations between factors. This pattern indicates that the factors are measuring distinct constructs rather than just different aspects of the same construct, providing strong evidence for discriminant validity.

## 4 Discussion

The results of the psychometric evaluation provide preliminary support for the structural, convergent and discriminant validity, as well as the reliability of the instrument. Additionally, all but one of the standardised factor loadings were significant and higher than the acceptance value of 0.50 (Hair, Babin, and Anderson 2018). The instrument also had good‐to‐excellent internal consistency reliability and CR for all subscales, with all higher than 0.80 (Field 2009). Therefore, these data support the instrument's potential as a valuable new tool for measuring family members' knowledge, contemplation, confidence and readiness regarding family engagement in early mobilisation for critically ill patients.

The instrument that we developed in this study is the first psychometrically supported questionnaire specifically assessing family members' knowledge, contemplation, confidence and readiness for engaging them in early mobilisation of adult critically ill patients. More broadly, Goldfarb et al. (2022) recently developed the Family Engagement (FAME) tool to measure family engagement, informed by literature, experts, family members and end‐users. The FAME tool contains 12 items that focus on family engagement, family centred care and perceptions of engagement (Goldfarb et al. 2022). While this tool provides an assessment of the extent to which family members are engaged, the FAME does not identify who and to what extent family members might be prepared to engage with care provision, including mobility exercise.

This newly developed instrument builds upon established theoretical concepts from Bandura (1977) and Prochaska and DiClemente (1983), emphasising the importance of family members' knowledge, contemplation, confidence and readiness to participate in early mobilisation. From these theories, we identified that family members first needed to know about early mobilisation (knowledge), before they could consider it (contemplation), gain confidence with it (self‐efficacy) and engage in this activity (readiness). Furthermore, the positive psychometric results support the instrument's use in both research and clinical settings. It offers insights that have the potential to improve our understanding of family members' knowledge, contemplation, confidence and readiness for engaging them in early mobilisation. Healthcare professionals can also utilise this survey to screen and assess individual family members' preparedness levels before engaging them in early mobilisation. The resulting knowledge, contemplation, confidence and readiness data can directly help healthcare professionals to inform the development of tailored strategies and/or interventions to effectively support individual family members become active partners in early mobilisation activities across critical care settings.

Although the new instrument demonstrated a good structural, convergent and discriminant validity and good‐to‐excellent internal consistency, there was low variation among the current sample on certain items, such as item one. According to the data distribution (File S3), item one of the knowledge factor had a truncated distribution with negative skewness and positive kurtosis. Assessing individual responses revealed that most respondents primarily endorsed two response options, including ‘extremely’ and ‘fairly’ (File S4). These distribution weaknesses can negatively affect the standardised factor loading of item one (Hair, Babin, and Anderson 2018). Accordingly, future research could check the performance of item one in other demographic or potentially rewording it to make it sensitive to greater variation in responses.

It is crucial to note that the CFA model, based on a priori theoretical assumptions, demonstrated satisfactory fit without modification, unequivocally confirming the structural validity of the proposed factor structure. The subsequent exploration of correlated errors between items one and two, assessing pre‐ and during hospitalisation physical function, was purely supplementary. This exploration was not necessary for validating the instrument, as this had already been achieved with the original model. The high modification index for these items aligns with the theoretical expectation that respondents might rely on similar cognitive processes or reference points when answering conceptually overlapping questions, leading to shared variance beyond the ‘knowledge’ factor. This shared variance could be attributed to method variance (due to similar wording), conceptual overlap, or response bias. However, allowing these errors to correlate yielded negligible improvement in model fit. While the chi‐square difference test showed a decrease, it was not significant at the conservative *α* = 0.001 often used in CFA (Hu and Bentler 1999). The primary fit indices remained excellent, further supporting the robustness of the original model. This exploratory modification offers insights into potential sources of shared variance, refining our understanding of the data and informing future scale development, but does not alter the fundamental validity of the instrument.

### 4.1 Limitations

This study has several limitations. Firstly, this study was conducted during the COVID‐19 pandemic amid a state lockdown and a visiting restriction at the hospitals. Consequently, family members' perspectives of engagement in care and early mobilisation activities for critically ill patients may have been influenced by the pandemic, which may affect their answers in the survey. Secondly, nearly half of the respondents were not living with the family member that was being treated in the ICU and this might substantially impact family dynamics and, consequently, influence their desire and willingness to engage in early mobilisation.

Thirdly, the instrument included ‘not sure’ as a response option, which did not fit conceptually on the underlying continuum represented by the response scale. For this reason, ‘not sure’ responses were treated as missing data in the analysis. Although occurrence of ‘not sure’ responses in each item was low, this may have affected the data distribution and the results. Accordingly, to assess the potential influence of this, it might be useful to remove the ‘not sure’ response for future validation of this instrument. Finally, the high reliability estimates and factor loadings raise concerns about multicollinearity and suggest potential redundancy among items. While CR values may be slightly overestimated with a small number of items per factor, the more conservative Omega (*ω*) estimates remain within a reasonable range. However, despite distinct semantic content and the importance of each item for content validity, the high reliability estimates and factor loadings for some items warrant further exploration in future research. Additionally, though referencing different targets, overly similar phrasing among items (e.g., *‘…ask questions of the therapist regarding the mobility and rehabilitation care of your family member?’* and *‘…ask questions of the nurse regarding the mobility and rehabilitation care of your family member?’*) may overestimate internal consistency and create response patterns resulting from participants not sufficiently differentiating between them. Future exploration would benefit from examining rephrased versions of these items in a new sample or using Rasch local dependency tests to further refine the instrument.

### 4.2 Recommendations for Further Research

The instrument was developed and evaluated through psychometric testing with 370 family members who were able to complete this survey in English across diverse geographical and socioeconomic areas within ICU settings across health services at southeast, Queensland. Although the concept of family engagement has cultural foundations, the factors of family members regarding their engagement in early mobilisation were not yet explored in the current study. As a result, our developed instrument requires further research to assess the applicability of the survey in other languages and cultures and across different ICU settings or areas of critical care to enhance the practice of family engagement in early mobilisation. Because of the asymmetrical data distribution, a larger sample size is recommended for future psychometric testing of the instrument to enhance the reliability and generalisability of the results. Additionally, the modified model, which allows for correlated errors between items one and two, requires further validation in independent samples to ensure it is not a sample‐specific artefact. If future analyses consistently support the estimation of these correlated errors, it may indicate the need to revise the wording or content of items one and two to reduce redundancy and enhance the distinctiveness of the measured constructs. Future research should also evaluate additional types of validity evidence, such as convergent and discriminant validity with other instruments, as well as criterion validity with key outcomes such as engagement intentions and behaviour.

### 4.3 Implications for Policy and Practice

This study presents support for the psychometric quality of the new instrument, which has the potential to help identify family members who are receptive to partnering with healthcare professionals in early mobilisation activities for critically ill patients. The findings hold promise for helping healthcare professionals pinpoint intervention targets by understanding family members' knowledge, contemplation, confidence and readiness regarding their engagement in early mobilisation.

## 5 Conclusion

The current results provide promising evidence for the structural, convergent and discriminant validity as well as reliability of a newly developed instrument measuring family members' knowledge, contemplation, confidence and readiness to engage in early mobilisation for critically ill patients. Its comprehensive approach offers a strategic guide for healthcare teams to identify areas for improvement, enabling tailored interventions that enhance patient recovery and family satisfaction. Further research should aim to corroborate these results and refine the instrument's response options as well as explore local dependency among items. Adapting the instrument to various languages, ICUs and clinical settings will strengthen its utility and broaden its applicability. Ultimately, this instrument offers a valuable opportunity to cultivate meaningful relationships between healthcare teams and family members, leading to improved patient recovery through personalised support strategies.

## Author Contributions

**Sasithorn Mukpradab** contributed to the study conceptualisation and methodology, project administration, investigation, formal analysis, validation, data curation, writing‐original draft, writing‐review & editing and visualisation. **Michael Ireland** contributed to formal analysis, validation, data curation, visualisation, writing‐review & editing. **Daren Heyland** contributed to the instrument development, the study conceptualisation and methodology, investigation and writing‐review & editing. **Andrea Marshall and Kristen Ranse** contributed to the study conceptualisation and methodology, investigation, project administration, resources, formal analysis, validation, visualisation, writing‐review & editing and supervision. **Georgia Tobiano, Fiona Coyer and Kellie Sosnowski** contributed to investigation, project administration, resources and writing‐review & editing.

## Disclosure

In kind resources were provided by Gold Coast Health, Princess Alexandra Hospital, Royal Brisbane and Women's Hospital and Logan Hospital.

## Conflicts of Interest

The authors declare no conflicts of interest.

### Peer Review

The peer review history for this article is available at https://www.webofscience.com/api/gateway/wos/peer‐review/10.1111/jan.16415.

## Supporting information


File S1.

File S2.

File S3.

File S4.

## Data Availability

Owing to the conditions under which participant consent was gained and the requirements of the institution providing ethical approval, only named researchers on the ethics application are permitted access to the raw data.
