# Supplementary material for: Psychometric Testing of an Instrument Assessing Family Knowledge, Contemplation, Confidence and Readiness for Engaging in Early Mobilisation of Critically Ill Patients: A Multi‐Site Cross‐Sectional Design
Source: J Adv Nurs. 2024 Sep 3;81(5):2382–92. doi: 10.1111/jan.16415 (PMC11967298; doi:10.1111/jan.16415)
Supplement: Supplementary file 1 — File S1. [file JAN-81-2382-s001.docx]

**Supplementary File 1**

**An Instrument Assessing Family Knowledge, Contemplation, Confidence, and Readiness for Engaging in Early Mobilisation of Critically ill Patients**

Now we want to talk about your family member’s mobilisation and participation in physical rehabilitation while in the ICU and hospital. Most patients are able to safely participate in mobility activities and physical rehabilitation during their critical illness with the help of therapists, nurses and others, including family members. This brief survey is designed to allow us to understand better your knowledge, confidence and readiness to help your family member to mobilise and participate in physical rehabilitation while in the ICU and hospital.

Please give us your honest opinions. There are no right or wrong answers.

| **Knowledge** |
| --- |

**These first questions ask about your understanding of mobility and rehabilitation in hospital.**

| **How well informed are you about…** | Not at all | A little | Somewhat | Fairly | Extremely | Not sure |
| --- | --- | --- | --- | --- | --- | --- |
| 1. The overall physical function of your family member prior to hospitalisation (i.e., walking ability, activity level, etc.)? | 1 | 2 | 3 | 4 | 5 | 8 |
| 1. The physical function and activity level of your family member during the current hospitalisation? | 1 | 2 | 3 | 4 | 5 | 8 |
| 1. The harms of inactivity and bed rest? | 1 | 2 | 3 | 4 | 5 | 8 |
| 1. The various mobility and rehabilitation treatment options in general? (Exercises, sitting out of bed, balance training, thinking activities, orientation, breathing exercises, standing and walking)? | 1 | 2 | 3 | 4 | 5 | 8 |
| 1. The mobility and rehabilitation care plan for your family member? | 1 | 2 | 3 | 4 | 5 | 8 |
| 1. The questions to ask about the mobility and rehabilitation care provided to your family member? | 1 | 2 | 3 | 4 | 5 | 8 |

| **Though about it (Contemplation)** |
| --- |

**These questions ask about how much you have thought about something.**

| **How much have you thought about…** | Never | Once or twice | A few times | Several times | A lot | Not sure |
| --- | --- | --- | --- | --- | --- | --- |
| 1. The importance of mobility and rehabilitation in the hospital? | 1 | 2 | 3 | 4 | 5 | 8 |
| 1. Seeking additional information on mobility and rehabilitation as it relates to the care of your family member? | 1 | 2 | 3 | 4 | 5 | 8 |
| 1. Asking questions of the therapists about the mobility and rehabilitation care of your family member? | 1 | 2 | 3 | 4 | 5 | 8 |
| 1. Asking questions of the nurse about the mobility and rehabilitation care of your family member? | 1 | 2 | 3 | 4 | 5 | 8 |
| 1. Asking questions of the doctor about the mobility and rehabilitation care of your family member? | 1 | 2 | 3 | 4 | 5 | 8 |
| 1. Participating in the mobility and rehabilitation care of your family member while in the hospital? | 1 | 2 | 3 | 4 | 5 | 8 |

| **Confidence (Self-efficacy)** |
| --- |

**These next questions ask about how confident you are to participate in the mobility and physical rehabilitation of your family member.**

| **How confident are you that today you could….** | Not at all | A little | Somewhat | Fairly | Extremely | Not sure |
| --- | --- | --- | --- | --- | --- | --- |
| 1. Seek out additional information on mobility and rehabilitation as it relates to the care of your family member? | 1 | 2 | 3 | 4 | 5 | 8 |
| 1. Ask questions of the therapist regarding the mobility and rehabilitation care of your family member? | 1 | 2 | 3 | 4 | 5 | 8 |
| 1. Ask questions of the nurse regarding the mobility and rehabilitation care of your family member? | 1 | 2 | 3 | 4 | 5 | 8 |
| 1. Ask questions of the doctor regarding the mobility and rehabilitation care of your family member? | 1 | 2 | 3 | 4 | 5 | 8 |
| 1. Participate in the mobility activity of your family member while in the hospital? | 1 | 2 | 3 | 4 | 5 | 8 |

1. If you lack confidence in doing one or more of the activities above, please explain why:

|  |
| --- |
|  |
|  |
|  |
|  |

| **Readiness (and stage of completion)** |
| --- |

| 1. How ready are you to seek out additional information on mobility and rehabilitation as it relates to the care of your family member?   1 🞎 I have never thought about it  2 🞎 I have thought about it, but I am not ready to do it   \| Why not? \| \| --- \| \|  \| \|  \|   3 🞎 I am thinking about doing it during this hospitalisation  4 🞎 I am definitely planning to do it during this hospitalisation  5 🞎 I have already done it | 8🞎 Not sure |
| --- | --- | --- | --- | --- |
| 1. How ready are you to talk with a therapist and ask questions about the mobility and rehabilitation care of your family member?   1 🞎 I have never thought about it  2 🞎I have thought about it, but I am not ready to do it   \| Why not? \| \| --- \| \|  \| \|  \|   3 🞎 I am thinking about doing it during this hospitalisation  4 🞎 I am definitely planning to do it during this hospitalisation  5 🞎 I have already done it | 8🞎Not sure |
| 1. How ready are you to talk with a nurse and ask questions about the mobility and rehabilitation care of your family member?   1 🞎 I have never thought about it  2 🞎 I have thought about it, but I am not ready to do it   \| Why not? \| \| --- \| \|  \| \|  \|   3 🞎 I am thinking about doing it during this hospitalisation  4 🞎 I am definitely planning to do it during this hospitalisation  5 🞎 I have already done it | 8🞎 Not sure |
| 1. How ready are you to talk with a doctor and ask questions about the mobility and rehabilitation care of your family member?   1 🞎 I have never thought about it  2 🞎 I have thought about it, but I am not ready to do it   \| Why not? \| \| --- \| \|  \| \|  \|   3 🞎 I am thinking about doing it during this hospitalisation  4 🞎 I am definitely planning to do it during this hospitalisation  5 🞎 I have already done it | 8🞎 Not sure |
| 1. How ready are you to participate in the mobility activity of your family member in while in the hospital?   1 🞎 I have never thought about it  2 🞎I have thought about it, but I am not ready to do it   \| Why not? \| \| --- \| \|  \| \|  \|   3 🞎 I am thinking about doing it during this hospitalisation  4 🞎 I am definitely planning to do it during this hospitalisation  5 🞎 I have already done it | 8🞎Not sure |

1. Please tell us about anything that prevents or prevented you from actively engaging in the mobility and rehabilitation care of your family member while hospitalized.

|  |
| --- |
|  |
|  |
|  |
|  |

Overall, how confident you are to partner with a member of the health care team to assist your loved one recover from their critical illness?

9 0

8 0

7 0

6 0

5 0

4 0

3 0

2 0

1 0

100

Not at all

0

Extremely confident
