# Supplementary material for: Psychometric Testing of an Instrument Assessing Family Knowledge, Contemplation, Confidence and Readiness for Engaging in Early Mobilisation of Critically Ill Patients: A Multi‐Site Cross‐Sectional Design
Source: J Adv Nurs. 2024 Sep 3;81(5):2382–92. doi: 10.1111/jan.16415 (PMC11967298; doi:10.1111/jan.16415)
Supplement: Supplementary file 2 — File S2. [file JAN-81-2382-s002.docx]

**Supplementary file 2**

The standardised factor loading in each item and the AVE and CR for each set of subscale items of the instrument before the model modification (N = 370)

| Items | Error variance | The initial stipulation | | |
| --- | --- | --- | --- | --- |
|  |  | The standardised factor loading^*^ | AVE | CR |
| Knowledge |  |  | 0.64 | 0.92 |
| Q1 | 0.23 | 0.49 |  |  |
| Q2 | 0.28 | 0.66 |  |  |
| Q3 | 0.32 | 0.82 |  |  |
| Q4 | 0.35 | 0.89 |  |  |
| Q5 | 0.33 | 0.91 |  |  |
| Q6 | 0.34 | 0.91 |  |  |
| Thought about it (contemplation) | | | 0.78 | 0.97 |
| Q7 | 0.13 | 0.81 |  |  |
| Q8 | 0.17 | 0.89 |  |  |
| Q9 | 0.18 | 0.96 |  |  |
| Q10 | 0.17 | 0.94 |  |  |
| Q11 | 0.16 | 0.90 |  |  |
| Q12 | 0.13 | 0.78 |  |  |
| Confidence (self-efficacy) | | | 0.83 | 0.96 |
| Q13 | 0.19 | 0.93 |  |  |
| Q14 | 0.19 | 0.95 |  |  |
| Q15 | 0.20 | 0.97 |  |  |
| Q16 | 0.18 | 0.93 |  |  |
| Q17 | 0.12 | 0.76 |  |  |
| Readiness | | | 0.71 | 0.93 |
| Q18 | 0.27 | 0.88 |  |  |
| Q19 | 0.28 | 0.90 |  |  |
| Q20 | 0.27 | 0.88 |  |  |
| Q21 | 0.24 | 0.81 |  |  |
| Q22 | 0.22 | 0.74 |  |  |

Note: * *p* < .001. Error variance is 1- *α*^2^.
