# Supplementary material for: Psychometric Testing of an Instrument Assessing Family Knowledge, Contemplation, Confidence and Readiness for Engaging in Early Mobilisation of Critically Ill Patients: A Multi‐Site Cross‐Sectional Design
Source: J Adv Nurs. 2024 Sep 3;81(5):2382–92. doi: 10.1111/jan.16415 (PMC11967298; doi:10.1111/jan.16415)
Supplement: Supplementary file 3 — File S3. [file JAN-81-2382-s004.docx]

**Supplementary File 3**

**The data distribution of each question item**

| **Items** | **Descriptive statistics** | | | | |
| --- | --- | --- | --- | --- | --- |
|  | **Variance statistics** | **Skewness** | | **Kurtosis** | |
|  |  | **Statistics** | **Standard error** | **Statistics** | **Standard error** |
| **Knowledge** |  |  |  |  |  |
| 1. The overall physical function of your family member prior to hospitalisation (i.e., walking ability, activity level, etc.)? | 0.876 | -2.091 | 0.129 | 3.909 | 0.257 |
| 2. The physical function and activity level of your family member during the current hospitalisation? | 1.293 | -1.105 | 0.129 | 0.426 | 0.257 |
| 3. The harms of inactivity and bed rest? | 1.385 | -1.227 | 0.129 | 0.573 | 0.257 |
| 4. The various mobility and rehabilitation treatment options in general? (Exercises, sitting out of bed, balance training, thinking activities, orientation, breathing exercises, standing, and walking)? | 1.859 | -0.701 | 0.130 | -0.772 | 0.259 |
| 5. The mobility and rehabilitation care plan for your family member? | 2.301 | -0.114 | 0.131 | -1.457 | 0.262 |
| 6. The questions to ask about the mobility and rehabilitation care provided to your family member? | 1.884 | -0.510 | 0.131 | -0.929 | 0.262 |
| **Thought about it (contemplation)** |  |  |  |  |  |
| 7. The importance of mobility and rehabilitation in the hospital? | 1.469 | -0.845 | 0.129 | -0.405 | 0.257 |
| 8. Seeking additional information on mobility and rehabilitation as it relates to the care of your family member? | 1.978 | -0.362 | 0.129 | -1.176 | 0.257 |
| 9. Asking questions of the therapists about the mobility and rehabilitation care of your family member? | 2.121 | -0.391 | 0.130 | -1.223 | 0.259 |
| 10. Asking questions of the nurse about the mobility and rehabilitation care of your family member? | 2.053 | -0.316 | 0.129 | -1.228 | 0.257 |
| 11. Asking questions of the doctor about the mobility and rehabilitation care of your family member? | 2.228 | -0.134 | 0.129 | -1.405 | 0.258 |
| 12. Participating in the mobility and rehabilitation care of your family member while in the hospital? | 2.138 | -0.554 | 0.132 | -1.097 | 0.264 |
| **Confidence (self-efficacy)** |  |  |  |  |  |
| 13. Seek out additional information on mobility and rehabilitation as it relates to the care of your family member? | 0.984 | -1.048 | 0.129 | 0.396 | 0.258 |
| 14. Ask questions of the therapist regarding the mobility and rehabilitation care of your family member? | 1.009 | -1.367 | 0.130 | 1.313 | 0.259 |
| 15. Ask questions of the nurse regarding the mobility and rehabilitation care of your family member? | 0.873 | -1.218 | 0.129 | 1.055 | 0.258 |
| 16. Ask questions of the doctor regarding the mobility and rehabilitation care of your family member? | 1.185 | -1.186 | 0.130 | 0.698 | 0.259 |
| 17. Participate in the mobility activity of your family member while in the hospital? | 1.169 | -1.292 | 0.130 | 0.915 | 0.259 |
| **Readiness (and stage of completion)** |  |  |  |  |  |
| 18. How ready are you to seek out additional information on mobility and rehabilitation as it relates to the care of your family member? | 1.414 | -0.794 | 0.134 | -0.133 | 0.267 |
| 19. How ready are you to talk with a therapist and ask questions about the mobility and rehabilitation care of your family member? | 1.272 | -0.866 | 0.134 | 0.229 | 0.266 |
| 20. How ready are you to talk with a nurse and ask questions about the mobility and rehabilitation care of your family member? | 1.128 | -0.849 | 0.133 | 0.474 | 0.265 |
| 21. How ready are you to talk with a doctor and ask questions about the mobility and rehabilitation care of your family member? | 1.294 | -0.815 | 0.133 | 0.115 | 0.265 |
| 22. How ready are you to participate in the mobility activity of your family member in while in the hospital? | 1.153 | -0.780 | 0.134 | 0.285 | 0.266 |
