# Supplementary material for: Psychometric Testing of an Instrument Assessing Family Knowledge, Contemplation, Confidence and Readiness for Engaging in Early Mobilisation of Critically Ill Patients: A Multi‐Site Cross‐Sectional Design
Source: J Adv Nurs. 2024 Sep 3;81(5):2382–92. doi: 10.1111/jan.16415 (PMC11967298; doi:10.1111/jan.16415)
Supplement: Supplementary file 4 — File S4. [file JAN-81-2382-s003.docx]

**Supplementary File 4**

**The response distribution to survey items (N = 370)**

| **Items** | **1** | **2** | **3** | **4** | **5** |
| --- | --- | --- | --- | --- | --- |
| **Knowledge^a^** |  |  |  |  |  |
| 1. How well informed are you about the overall physical function of your family member prior to hospitalisation (i.e., walking ability, activity level, etc.)? | 8(2.20%) | 14(3.80%) | 22(5.90%) | 62(16.80%) | 254(68.60%) |
| 2. How well informed are you about the physical function and activity level of your family member during the current hospitalisation? | 17(4.60%) | 25(6.80%) | 51(13.80%) | 107(28.90%) | 159(43.00%) |
| 3. How well informed are you about the harms of inactivity and bed rest? | 20(5.40%) | 24(6.50%) | 43(11.60%) | 93(25.1%) | 179(48.40%) |
| 4. How well informed are you about the various mobility and rehabilitation treatment options in general? (Exercises, sitting out of bed, balance training, thinking activities, orientation, breathing exercises, standing, and walking)? | 40(10.80%) | 41(11.10%) | 48(13.00%) | 98(26.50%) | 125(33.80%) |
| 5. How well informed are you about the mobility and rehabilitation care plan for your family member? | 89(24.10%) | 45(12.20%) | 54(14.60%) | 79(21.40%) | 78(21.10%) |
| 6. How well informed are you about the questions to ask about the mobility and rehabilitation care provided to your family member? | 52(14.10%) | 32(8.60%) | 73(19.70%) | 94(25.40%) | 94(25.40%) |
| **Though about it (contemplation)^b^** |  |  |  |  |  |
| 7. How much have you thought about the importance of mobility and rehabilitation in the hospital? | 16(4.30%) | 35(9.50%) | 70(18.90%) | 66(17.80%) | 170(45.90%) |
| 8. How much have you thought about seeking additional information on mobility and rehabilitation as it relates to the care of your family member? | 52(14.10%) | 56(15.10%) | 62(16.80%) | 88(23.80%) | 100(27.00%) |
| 9. How much have you thought about asking questions of the therapists about the mobility and rehabilitation care of your family member? | 59(15.90%) | 47(12.70%) | 60(16.20%) | 79(21.40%) | 109(29.50%) |
| 10. How much have you thought about asking questions of the nurse about the mobility and rehabilitation care of your family member? | 58(15.70%) | 52(14.10%) | 68(18.40%) | 78(21.10%) | 101(27.30%) |
| 11. How much have you thought about asking questions of the doctor about the mobility and rehabilitation care of your family member? | 80(21.60%) | 53(14.30%) | 61(16.50%) | 75(20.30%) | 86 (23.20%) |
| 12. How much have you thought about participating in the mobility and rehabilitation care of your family member while in the hospital? | 54(14.60%) | 38(10.30%) | 48(13.00%) | 80(21.60%) | 119(32.20%) |
| **Confidence^a^** |  |  |  |  |  |
| 13. How confident are you that today you could seek out additional information on mobility and rehabilitation as it relates to the care of your family member? | 5(1.40%) | 22(5.90%) | 55(14.90%) | 104(28.10%) | 171(46.20%) |
| 14. How confident are you that today you could ask questions of the therapist regarding the mobility and rehabilitation care of your family member? | 8(2.20%) | 20(5.40%) | 38(10.30%) | 102(27.60%) | 188(50.80%) |
| 15. How confident are you that today you could ask questions of the nurse regarding the mobility and rehabilitation care of your family member? | 5(1.40%) | 13(3.50%) | 52(14.10%) | 102(27.60%) | 186(50.30%) |
| 16. How confident are you that today you could ask questions of the doctor regarding the mobility and rehabilitation care of your family member? | 13(3.50%) | 20(5.40%) | 53(14.30%) | 95(25.70%) | 175(47.30%) |
| 17. How confident are you that today you could participate in the mobility activity of your family member while in the hospital? | 12(3.20%) | 20(5.40%) | 47(12.70%) | 86(23.20%) | 189(51.10%) |
| **Readiness^c^** |  |  |  |  |  |
| 18. How ready are you to seek out additional information on mobility and rehabilitation as it relates to the care of your family member? | 38(10.30%) | 20(5.40%) | 74(20.00%) | 141(38.10%) | 63(17.00%) |
| 19. How ready are you to talk with a therapist and ask questions about the mobility and rehabilitation care of your family member? | 33(8.90%) | 15(4.10%) | 82(22.20%) | 148(40.00%) | 60(16.20%) |
| 20. How ready are you to talk with a nurse and ask questions about the mobility and rehabilitation care of your family member? | 20(5.40%) | 13(3.50%) | 87(23.30%) | 130(35.10%) | 91(24.60%) |
| 21. How ready are you to talk with a doctor and ask questions about the mobility and rehabilitation care of your family member? | 33(8.90%) | 17(4.60%) | 86(23.20%) | 140(37.80%) | 65(17.60%) |
| 22. How ready are you to participate in the mobility activity of your family member in while in the hospital? | 27(7.30%) | 18(4.90%) | 95(25.70%) | 142(38.40%) | 55(14.90%) |

Note: ^a^ 1 = Not at all, 2 = A little, 3 = Somewhat, 4 = Fairly, and 5 = Extremely

^b^ 1 = Never, 2 = Once or twice, 3 = A few times, 4 = Several times, and 5 = A lot

^c^ 1 = I have never thought about it, 2 = I have thought about it, but I am not ready to do it, 3 = I am thinking about doing it during this hospitalisation, 4 = I am definitely planning to do it during this hospitalisation, and 5 = I have already done it
